# Draft genome sequences of *Staphylococcus* species isolated from urine samples from asymptomatic females

**DOI:** 10.1128/mra.00812-25

**Published:** 2025-09-30

**Authors:** Sandra Jablonska, Lila Nelson, Grace Finger, Alex Kula, Catherine Putonti

**Affiliations:** 1 Bioinformatics Program, Loyola University Chicago 2456, Chicago, Illinois, USA; 2 Biology Department, Loyola University Chicago 224010, Chicago, Illinois, USA; Fluxus Inc., Sunnyvale, California, USA

**Keywords:** *Staphylococcus aureus*, *Staphylococcus haemolyticus*, *Staphylococcus hominis*, urinary microbiome

## Abstract

The female urinary tract is host to several different *Staphylococcus* species, both uropathogens and commensals. To further investigate these species, we sequenced the genomes of four isolates from voided urine samples collected from asymptomatic (“healthy”) females: *Staphylococcus aureus* U215, *Staphylococcus haemolyticus* U117, and *Staphylococcus hominis* strains U224 and U226.

## ANNOUNCEMENT

Although typically associated with the skin and mucosa, *Staphylococcus* species also inhabit the urinary tract. While *Staphylococcus aureus* is a known uropathogen, it is not prevalent (1). However, coagulase-negative staphylococci species are common, in particular in the urine of females with urge urinary incontinence (2, 3). While some species are more frequently found, e.g., *Staphylococcus epidermidis* and *Staphylococcus haemolyticus*, others are rare, e.g., *Staphylococcus hominis* (2). Here, we present the draft genome assemblies of one *S*. *aureus*, one *S*. *haemolyticus*, and two *S*. *hominis* isolates from urine collected from four different self-identified “healthy” females who had not taken antibiotics within the last 6 months.

Participants in our institutional review board (IRB)-approved study (IRB no. 3603) were students at Loyola University Chicago (41.99872, −87.6577). Each participant was instructed to collect a first-morning, mid-stream urine sample. Within 1 hour of sampling, 100 µL of urine was spread on a mannitol salt (MS) agar plate and incubated for 24 hours at 35°C with 5% CO_2_. MS broth (1 mL) was inoculated with a morphologically distinct colony and incubated for 24 hours at 35°C with 5% CO_2_ without shaking. This process was repeated to purify the isolates. DNA was extracted using the DNeasy Blood and Tissue kit (Qiagen), following the manufacturer’s protocol for gram-positive bacteria. DNA was quantified via a Qubit fluorometer and then sent to SeqCoast (Portsmouth, NH USA) for library construction (Illumina DNA Prep tagmentation kit and unique dual indexes) and sequencing (Illumina NextSeq2000 platform; 2 × 150 bp reads). Prior to delivery from SeqCoast, DRAGEN v3.10.12 (Illumina) was used for read demultiplexing, read trimming, and run analytics.

Assembly was performed using the BV-BRC v3.51.7 web tool with the “auto” option (4). Raw reads were trimmed using trim_galore v0.6.5dev (https://github.com/FelixKrueger/TrimGalore), normalized via bbnorm v37.60 (https://sourceforge.net/projects/bbmap/), and assembled using Unicycler v0.4.8 (5), followed by two rounds of polishing with Pilon v1.24 (6). To determine the species of each isolate, taxonomic binning of raw reads was conducted via BV-BRC (which uses Kraken 2 [7]). The BV-BRC identified species was confirmed by JSpeciesWS (8), comparing each draft assembly to species representatives within the JSpeciesWS GenomesDB, and by NCBI upon submission. Genome annotations were generated by PGAP v6.10 (9). Completeness and contamination metrics were computed using CheckM v1.2.3 (10) upon submission to NCBI. Genomes were also examined for prophage sequences using PHASTER (11). Unless otherwise noted, default parameters were used for all software tools.

Table 1 lists the genome assembly statistics. While temperate phages of *S. aureus* have been shown to be significant drivers of the species’ evolution (12), the diversity of *Staphylococcus*-infecting phages has only recently been explored (13, 14). Intact prophage sequences were found in the *S. aureus* and *S. hominis* sequences. *S. hominis* U224 encoded for two phages, one of which is also present in the *S. hominis* U226 genome. Further characterization of *Staphylococcus*-infecting phages of the urinary tract is needed to ascertain their role in shaping these species for this environment.

**TABLE 1:** Genome assembly statistics for *S. aureus* U215, *S. haemolyticus* U117, and *S. hominis* U224 and U226

Strain	U215	U117	U224	U226
Species designation	*S. aureus*	*S. haemolyticus*	*S. hominis*	*S. hominis*
ANI (%)	98.07^*a*^	98.70^*b*^	98.58^*c*^	98.80^*c*^
Assembly length (bp)	2,738,746	2,435,287	2,293,153	2,398,823
No. of contigs	46	82	51	41
Contigs N50 (bp)	176,606	65,388	86,826	146,863
Coverage (×)	193.88	166.08	438.37	413.62
# genes	2,737	2,471	2,329	2,433
# tRNAs	56	55	50	55
G + C (%)	32.70	32.54	31.33	31.39
Completeness (%)	98.08	99.25	91.55	89.78
Contamination (%)	0.39	0.06	3.77	5.06
No. of reads	3,784,218	2,653,578	8,473,308	8,992,872
SRA accession no.	SRR34298188	SRR34547675	SRR34298187	SRR34298186
Assembly accession no.	JBPFAR000000000	JBPFAQ000000000	JBPFAS000000000	JBPFAT000000000

^*a*^ *S. aureus* 502A (GCF_000597965.1).

^*b*^ *S. haemolyticus* 1HT3 (GCF_000981145.1).

^*c*^ *S*. *hominis* 2842STDY5753564 (GCA_900033555.1).

## Data Availability

Accession numbers for the deposited raw reads and annotated assembly can be found in Table 1.
